# Nail disorders associated with cast immobilization of the forearm and wrist: report of two cases and review of the literature^☆^^☆☆^

**DOI:** 10.1016/j.abd.2020.07.024

**Published:** 2021-09-14

**Authors:** Sıla Kılıç Sayar, Yasin Sayar

**Affiliations:** a Department of Dermatology and Venereology, Bahçeşehir University Faculty of Medicine, Istanbul, Turkey; b Department of Orthopedics and Traumatology, Health Sciences University, Sultan II Abdülhamid Han Training and Research Hospital, Istanbul, Turkey

**Keywords:** Immobilization, Neoplasms, Pyogenic granuloma, Reflex sympathetic dystrophy

## Abstract

Cast immobilization is used in the management of various injuries of joints and/or limbs. A variety of nail disorders have been reported in association with cast immobilization of the forearm and wrist among a limited number of patients so far. The mechanism was not clearly identified in some of these cases. Here, the authors report two patients with nail disorders appeared after the removal of immobilization cast of forearm and wrist and review the literature.

## Introduction

Injuries including bone fractures, joint dislocations, and soft tissue lacerations of the hand, forearm, and wrist are common among all age groups.^1^ Immobilization cast, a device that covers the limb, is used to stabilize the joint and/or limb, control pain and support the healing of injured tissue.^1^ Nail disorders including nail fold edema, onychomadesis, pyogenic granuloma, and trachyonychia have been occasionally reported in association with the cast immobilization of the forearm and wrist.^2^, ^3^, ^4^, ^5^, ^6^, ^7^, ^8^, ^9^ The diagnosis is sometimes confusing particularly, in the cases that are free of neurological symptoms. The present authors aim to report two patients and describe the characteristics of other reported patients in whom nail disorders were seen in association with immobilization casts of the forearm and wrist.

## Case 1

**Figure 1:**
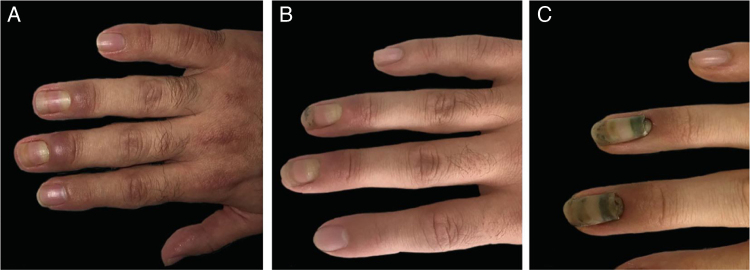
Clinical images of the first patient. (A), Prominent nail fold edema of the third and fourth fingers. (B), Regression of edema. (C), Onychomadesis in two fingers.

The first patient was a 28-year-old male who had a zone 2 flexor tendon laceration of the right thumb due to a perforating trauma. The tendon was surgically repaired and hand was immobilized with a cast that extended to the half of the forearm in the upper part and to the proximal nail folds of the second to fifth fingers in the lower part for three weeks. Two weeks after removal of the immobilization cast, a prominent periungual edema appeared on the third and fourth fingers of the same hand and resembled a bacterial whitlow (Fig. 1a). The patient was not describing any pain or other neurological symptoms. The neurological examination and electromyography (EMG) results were normal. Levels of the acute phase reactants and other basic laboratory markers were within the normal limits. Magnetic resonance imaging exhibited edema around the affected nails. Following the application of topical corticosteroids leading a poor clinical outcome, he was started on oral methylprednisolone (0.5 mg/kg/day) and tapered over two weeks. Edema resolved during the therapy (Fig. 1b) but onychomadesis appeared in the same two fingers (Fig. 1c).

## Case 2

**Figure 2:**
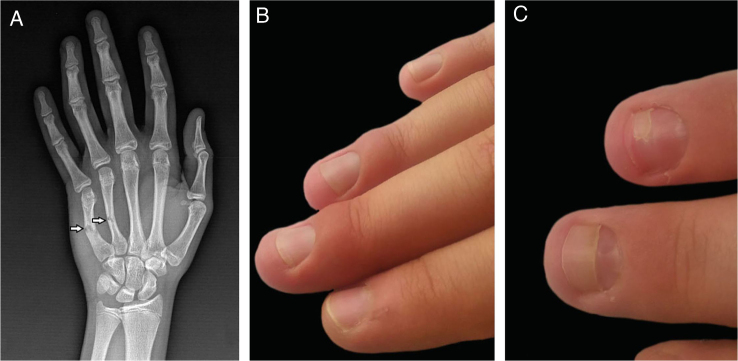
Clinical and radiological images of the second patient. (A), Fourth and fifth metacarpal bone fractures (X-Ray image). (B), Nail fold edema of the third and fourth fingers. (C), Onychomadesis in the two fingers.

The second patient was a 15-year-old male who had fractures of the fourth and fifth metacarpal bones in the right hand (Fig. 2a). The forearm, wrist, and hand were immobilized with an immobilization cast in intrinsic plus position. The cast was covering four fingers from the second to fifth and stayed there for three weeks. Two weeks after removal of the plaster cast, mild periungual edema appeared on the second to fourth fingers(Fig. 2b). The patient had no pain and neurological symptoms at the time of the first submission; however, he described mild tingling and pain during the immobilization. The neurological examination, EMG, and blood tests did not reveal any pathological results. Topical corticosteroids were applied and edema resolved in a few weeks, but onychomadesis occurred in the same two fingers (Fig. 2c).

## Discussion

A limited number of patients were reported with the nail disorders associated with the immobilization cast of the forearm and wrist.^2^, ^3^, ^4^, ^5^, ^6^, ^7^, ^8^, ^9^ Table 1 summarizes the published cases; including the 2 cases described here, a total of 20 patients (18 male and 2 female; age range, 15–45 years) were found in the literature.^2^, ^3^, ^4^, ^5^, ^6^, ^7^, ^8^, ^9^ Since the initial symptoms (mostly edema of the nail folds) appeared a while after the removal of the cast (range, a few days to six weeks), both dermatologists and orthopedists should be aware of this rare occurrence not to overlook the diagnosis.

**Table 1:** Characteristics of reported patients with nail disorders associated with the immobilization cast of the forearm and wrist.

Author (year)/Number of patients	Sex	Age (y)	Cause of the immobilization	Immobilization cast type and application details	Duration^a^	Nail findings/Fingers of the affected nails	Neurological signs and/or symptoms	Neurological examination/EMG results	Treatment	Course
Present authors (2020)/2 patients	M	28	Zone 2 flexor tendon laceration (first finger of the right hand)	Circumferential plaster cast (extending to 2^nd^–5^th^ fingers)	2 weeks	Nail fold edema, oncyhomadesis/3^rd^–4^th^	None	Normal/Normal	Systemic steroids, topical emolients	Complete healing
	M	15	Fracture of the 4^th^ and 5^th^ metacarpals (right hand)	Plaster cast in intrinsic plus position (entending to 4^th^–5^th^ fingers)	3 weeks	Nail fold edema, oncyhomadesis/3^rd^–4^th^	Local pain (during the immobilization)	Normal/Normal	Topical steroids	Complete healing
Baykal^2^ (2018)/1 patient	M	25	Fracture of the middle phalanx of the left 3^rd^ finger	Circumferencial plaster cast of forearm + wrist (extending to 2^nd^–5^th^ fingers)	3 weeks	PG, nail fold edema, oncyhomadesis/ 2^nd^–4^th^	None	Normal/Normal	Systemic steroid and antibiotics, topical antibiotics	Complete healing
Thakur^3^ (2016)/1 patient	M	14	Left distal radio-ulnar joint dislocation	Plaster cast from above the elbow up to the metacarpal bones	6 weeks	PG, oncyhomadesis/ 1^st^–2^nd^	Local increased sweating	Normal/Normal	Curettage	NA
Whitelaw^4^ (2014)/1 patient	F	10	Fracture of the proximal phalanx of left 1^st^ finger	Plaster cast extending to the hand and wrist (fingers mobile)	NA	Nail fold edema and oncyhomadesis/1^st^–3^rd^	None	NA	None	Complete healing
Pampin^5^ (2014)/1 patient	M	35	Distal detachment of the biceps brachii tendon	Plaster cast from halfway up the arm to metacarpal joint	2 weeks	PG and oncyhomadesis/2^nd^–4^th^ and 2^nd^–5^th^, respectively	None	Normal/Normal	None	Complete healing
Piraccini^6^ (2010)/3 patients	M	43/42/45	Wrist or other bone fracture of the limb	Plaster cast (non-detailed)	3 months^b^	PG/ NA	Severe pain, hyperhidrosis in one patient	Normal/Normal	Surgical removal in 1, silver nitrate treatment in 2 patients	Complete healing in two patients
Pucevich^7^ (2008)/1 patient	F	48	Fracture in the right 3rd finger	Plaster cast of forearm + wrist (non-detailed)	NA	Trachyonychia/All fingers of a single hand	Pain, motor loss and dysesthesia of the arm	NA	Moisturizer, alpha-acetoxyacid, oral biotin	NA
Tosti^8^ (2001)/9 patients	M	15–42	Fracture of a phalanx in 3, a metacarpal bone in 2, and the wrist in 4 patients	Cast of forearm + wrist (non-detailed)	7–30 days	PG in all, oncyhomadesis in 3 patients/Not detailed	Moderate paresthesia and pain (during the immobilization)	Normal/Normal	NA	Complete healing
Tosti^9^ (1993)/1 patient	M	17	Ulnar-carpal joint fracture and ulnar collateral ligament damage	Plaster cast of forearm + wrist (non-detailed)	A few days	Nail fold edema/3^rd^–4^th^	Tingling, burning pain, hyperhidrosis and coldness	Normal/Normal	Antibiotic creams and systemic anti-inflammatoires	NA

EMG, Electromyelography; M, Male; F, Female; PG, Pyogenic Granuloma; NA, Not Applicable.

a The time between the removal of cast and the appearance of nail findings.

b The time between the injury and the appearance of nail findings.

While pain and neurological symptoms were absent in 14 of the reported patients, various symptoms developed after the removal of the cast in the other six patients.^2^, ^3^, ^4^, ^5^, ^6^, ^7^, ^8^, ^9^ These were as follows: burning pain, paresthesia, dysesthesia, hyperhidrosis, coldness, and tingling. Ten patients including all series of Tosti et al.^8^ and the study’s second patient were reported to complain of moderate paresthesia and pain during the cast immobilization. Although neurological examination and EMG results remained normal in all reported patients whose neurological examination data was applicable (n = 18), three patients were diagnosed with RSD: two patients by X-ray examination (patchy osteoporosis) and one patient by clinical diagnosis.^6^, ^7^, ^9^ The skin of the affected hand was reported to be cold, smooth, and shiny in one patient with RSD, and glossy, edematous, and scleroderma-like with sparse hair in the other.^7^, ^9^ Moreover, the latter patient's right forearm was described as with absent hair growth comparing to the left forearm.^7^

The diagnosis of RSD, a complex disorder reflecting a severe peripheral nerve injury, usually depends on the defined clinical findings of the patients because the results of laboratory tests and neurologic evaluation are normal in most cases.^7^, ^9^ Nail disorders during RSD were not unusual and they were also reported due to the different causes besides the cast immobilization.^10^ On the other hand, pressure-induced nerve injury (temporary and probably less severe than RSD) caused by the immobilization cast was held responsible for the short break of the nail growth in the patients without typical findings of RSD.^3^, ^4^, ^5^ This hypothesis suggests that the newly growing nail causes inflammation around the nails resembling a bacterial whitlow. Although the mechanism of the pyogenic granulomas occurred in some of the patients could not be identified, it is probably due to theexcessive vascularization associated with the inflammation.^2^ Since nail changes can be also seen in the fingers which were not affected in the primary injury, it is more likely that compression of the cast immobilization was responsible for the break in the nail growth rather that the primary injury itself.^2^, ^3^, ^4^, ^6^, ^8^

In conclusion, the mechanism has not clearly identified in all nail disorders that were seen in association with the cast immobilization of the forearm and wrist. Nail disorders associated with the immobilization casts are probably more common than it is reported and easy to be overlooked during our daily practices. It is important not to misdiagnose the initial edema as bacterial whitlow to avoid unnecessary use of antibiotics. Since pyogenic granulomas appeared following the prominent periungual edema in some of the reported patients, the authors believe that the early use of systemic corticosteroids in selected cases may both treat the prominent edema and prevent the possible development of pyogenic granulomas.

## Financial support

None declared.

## Authors’ contributions

Sıla Kılıç Sayar: Conceptualization-equal, data curation-equal, investigation-equal, methodology-equal, supervision-lead, visualization-equal, writing-original draft-lead, writing-review & editing- equal.

Yasin Saya: Conceptualization- equal, data curation-equal, investigation-equal, methodology-equal, supervision-supporting, visualization-equal, writing-original draft-supporting, writing-review & editing-equal.

## Conflicts of interest

None declared.
